# Pain management practice patterns after hip arthroscopy: an international survey

**DOI:** 10.1093/jhps/hnaa050

**Published:** 2020-12-30

**Authors:** Flávio L Garcia, Brady T Williams, Bhargavi Maheshwer, Asheesh Bedi, Ivan H Wong, Hal D Martin, Shane J Nho, Jorge Chahla

**Affiliations:** 1 Section of Young Adult Hip Surgery, Division of Sports Medicine, Department of Orthopedic Surgery, Rush Medical College of Rush University, Rush University Medical Center, 1611 W Harrison St, Chicago, IL, 60612, USA; 2 Department of Orthopaedic Surgery, Instituto Brasil de Tecnologias da Saúde, Rio de Janeiro, RJ, Brazil, Rua Visconde de Pirajá, 407 Rio de Janeiro, RJ, 22410-003, Brazil; 3 Department of Orthopaedics and Anesthesiology, Ribeirão Preto Medical School of the University of São Paulo, Ribeirão Preto, Avenida Bandeirantes, 3900 Ribeirão Preto, SP, 14049-900, Brazil; 4 Department of Orthopaedic Surgery, University of Michigan, 1500 E Medical Center Drive Ann Arbor, MI, 48109, USA; 5 Department of Surgery, Dalhousie University, Halifax, 6299 South St Halifax, NS, B3H 4R2, Nova Scotia, Canada; 6 Hip Preservation, aylor University Medical Center, 411 N Washington Ave, Suite 7300 Dallas, TX, 75246, USA

## Abstract

Several post-operative pain control methods have been described for hip arthroscopy including systemic medications, intra-articular or peri-portal injection of local anesthetics and peripheral nerve blocks. The diversity of modalities used may reflect a lack of consensus regarding an optimal approach. The purpose of this investigation was to conduct an international survey to assess pain management patterns after hip arthroscopy. It was hypothesized that a lack of agreement would be present in the majority of the surgeons’ responses. A 25-question multiple-choice survey was designed and distributed to members of multiple orthopedic professional organizations related to sports medicine and hip arthroscopy. Clinical agreement was defined as > 80% of respondents selecting a single answer choice, while general agreement was defined as >60% of a given answer choice. Two hundred and fifteen surgeons completed the survey. Clinical agreement was only evident in the use of oral non-steroidal anti-inflammatory drugs (NSAIDs) for pain management after hip arthroscopy. A significant number of respondents (15.8%) had to readmit a patient to the hospital for pain control in the first 30 days after hip arthroscopy in the past year. There is significant variability in pain management practice after hip arthroscopy. The use of oral NSAIDs in the post-operative period was the only practice that reached a clinical agreement. As the field of hip preservation surgery continues to evolve and expand rapidly, further research on pain management after hip arthroscopy is clearly needed to establish evidence-based guidelines and improve clinical practice.

## INTRODUCTION

Hip arthroscopy is exponentially growing worldwide as its indications continue to expand. As it is largely performed in an outpatient setting [1, 2], hip arthroscopy requires proper post-operative pain management. Adequate post-operative pain control is of utmost importance as it increases patient satisfaction, decreases opioid consumption and allows early ambulation and rehabilitation [3–5]. Moreover, inadequate post-operative pain control may lead to adverse physiologic effects, delayed recovery and increased risk of developing chronic pain associated with the procedure [6, 7].

Systematic reviews have demonstrated the efficacy of standardized strategies such as peripheral nerve blocks for pain management after knee [8] and shoulder [9] arthroscopy. In contrast, there is a paucity of high-quality comparative studies and an absence of standardized protocols for pain management after hip arthroscopy [10], making it difficult to determine the best approach for pain management post-operatively. Understanding the current practice patterns in analgesia after hip arthroscopy is imperative, as this may serve as a foundation for future establishment and improvement of perioperative practices for hip arthroscopy.

Several options for post-operative pain control have been described for hip arthroscopy including pre-emptive and post-operative systemic medications [3, 11], intra-articular or peri-portal injection of local anesthetics [12, 13] and peripheral nerve blocks, such as lumbar plexus block [14], fascia iliaca block [15] and femoral nerve block [16]. The diversity of modalities described may reflect a lack of consensus on an optimal approach to pain management after hip arthroscopy. The purpose of this investigation was to design and conduct an international survey analyzing the pain management practice patterns after hip arthroscopy. It was hypothesized that there would be a lack of clinical agreement in the majority of the surgeons’ responses regarding perioperative pain management.

## MATERIALS AND METHODS

### Study design and administration

The authors conducted a cross-sectional international survey of current practices of orthopedic surgeons in the management of pain following hip arthroscopy. For this investigation, Institutional Review Board approval was not required. The investigation did not include any patient data, and all survey data were collected and stored anonymously.

The questionnaire (Table I) consisted of 25 close-ended, multiple-choice questions that were divided into two categories: surgeon demographics, training and practice (10 questions) and pain management routine (15 questions).

**Table I.** Survey questions administered to participants

1. What is your current age?
2. To which professional organizations do you belong (mark all that apply)?
3. In what region of the world do you reside and practice?
4. How long have you been in practice?
5. Which of the following best describes your current practice environment?
6. Are you fellowship trained?
7. Did you receive formal training in hip arthroscopy during your fellowship?
8. Did you receive formal training in hip arthroscopy during your residency?
9. What is your main area of practice?
10. How many hip arthroscopies do you perform per year?
11. Do you routinely use pre-emptive analgesia (e.g. medications administered in preoperative area 1–2 h before the operation) prior to hip arthroscopy?
12. Which of the following medications do you routinely use for pre-emptive analgesia prior to hip arthroscopy? (select all that apply)
13. What is the primary factor that influences your choice for pre-emptive analgesia?
14. What is your standard pain management protocol after hip arthroscopy? (select all that apply)
15. What is the primary factor that influences your choice for your standard pain management protocol after hip arthroscopy?
16. Which of the following medications do you routinely use for intra-articular or soft tissue (peri-portal) anesthetic injection after hip arthroscopy? (select all that apply)
17. What is the primary factor that influences your choice for intra-articular or soft tissue (peri-portal) anesthetic injection after hip arthroscopy
18. What is your standard duration of treatment with oral medication for pain after hip arthroscopy?
19. What is your standard discharge time after hip arthroscopy?
20. How often do you provide verbal counseling on postoperative pain control after hip arthroscopy?
21. How often do you provide written instructions on postoperative pain control after hip arthroscopy?
22. How often do your patients report poor pain control after hip arthroscopy?
23. In the past year, how often have you referred a patient to a pain management specialist in the first 30 days after a hip arthroscopy?
24. In the past year, how often have you readmitted a patient to hospital for pain control in the first 30 days after a hip arthroscopy?
25. Has any opinion manuscript or expert consensus on clinical/surgical practice affected or directed your care of a patient or patient condition?

The survey was conducted using the Microsoft Forms application (Microsoft, Redmond, WA, USA). A link to the survey was distributed to all members of the following orthopedic societies: American Orthopaedic Society for Sports Medicine (AOSSM); European Society of Sports Traumatology, Knee Surgery and Arthroscopy (ESSKA); International Society for Hip Arthroscopy (ISHA); Brazilian Hip Society (SBQ); and Latin American Society of Arthroscopy, Knee and Sports (SLARD). The survey was sent to members per standard practices of each participating professional society, without additional follow-up emails to solicit completion. Participation was voluntary, and data were collected between November 2019 and April 2020.

### Data analysis

Responses to all questions were collected and tabulated. In accordance with previously published survey studies [17–20], statistical analyses were not performed. Survey respondents were subsequently divided and analyzed by geographic region to determine differences in regional preferences in pain management after hip arthroscopy.

### Clinical agreement

While the survey did not use questions requiring participants to respond ‘Agree’ or ‘Disagree’, for the purposes of this study and following previously published work [17, 21, 22], ‘clinical agreement’ was defined as >80% agreement in survey response and ‘general agreement’ as >60% agreement.

## RESULTS

### Characteristics of participants

Responses were collected from a total of 215 participants. A plurality of (41.8%) of participants were between the age of 41–50 years old (Table II), while the majority of the participants were affiliated to the International Society for Hip Arthroscopy (ISHA) (64.3%) (Table II). The greatest number of participants reported practicing in Central/South America (36.7%), followed by North America (31.2%) and Europe (25.6%) (Table II). The majority of the participants (61.6%) have been in practice for more than 11 years (Table II), with most in private practice (47.9%) or academic medicine (17.4%) (Table II). Over 83% of participants were fellowship-trained (Table II), and 57% of participants received formal training in hip arthroscopy during fellowship (Table II). In contrast, only 28% of respondents received formal training in hip arthroscopy during residency (Table II), with most of these responses coming from North America respondents (47% of North American respondents). With regards to specialty, 56.3% of reported sports medicine as his or her primary practice, followed by total joint replacement (38.5% of respondents) (Table II).

**Table II.** Surgeon profile and demographic information

What is your current age?	RR (%)	To which professional organizations do you belong? (select all that apply)	RR (%)	In what region of the world do you reside and practice?	RR (%)	How long have you been in practice?	RR (%)	Which of the following best describes your current practice environment?	RR (%)	Are you fellowship trained?	RR (%)	Did you receive formal training in hip arthroscopy during fellowship?	RR (%)	Did you receive formal training in hip arthroscopy during residency?	RR (%)	What is your main area of practice?	RR (%)	How many hip arthroscopies do you perform per year?	RR (%)
<30 years	1	ISHA	64.3	South/Central America	36.7	Less than 5 years	12.2	Private	47.9	Yes, multiple fellowships (2 or more)	36.6	Yes	57	Yes	28	Sports medicine	56.3	< 20	22.1
30–40 years	34.3	AANA	32.4	North America	31.2	5–10 years	26.3	Academic	17.4	Yes, 1 fellowship	46.9	No	43	No	72	Joints	38.5	20–50	22.5
41–50 years	41.8	AOSSM	26.3	Europe	25.6	11–15 years	25.4	Private/Academic	17.8	No	16.4					Pediatric orthopedics	3.3	51–100	24.4
51–60 years	19.7	SBQ	23.5	Asia/Pacific	4.2	16–20 years	15.5	Hospital Employee	15.0							Trauma	0.9	101–200	18.8
>60 years	3.3	ESSKA	18.8	Middle East/Africa	1.4	Greater than 20 years	20.7	Other	1.9							Foot and ankle	0.5	>200	12.2
		SLARD	11.3													Upper extremity	0.5		
		Other	22.5																

RR, response rate; ISHA, International Society for Hip Arthroscopy; AANA, Arthroscopy Association of North America; AOSSM, American Orthopaedic Society of Sports Medicine; SBQ, Brazilian Hip Society; ESSKA, European Society for Sports Traumatology, Knee Surgery, and Arthroscopy; SLARD, Latin American Society of Arthroscopy, Knee, and Sports.

### Hip arthroscopy and pain management

Approximately 70% of responding surgeons reported performing less than 100 hip arthroscopies per year (Table II). Pre-emptive analgesia was not routinely used by 41.3% of the respondents. Of those who utilized pre-emptive analgesia, the most common medications were non-steroidal anti-inflammatory drugs (NSAIDs) and non-opioid analgesics, such as acetaminophen or salicylates (Table III). The primary factor reported to influence the choice of pre-emptive analgesia was most commonly anesthesiologist preference (45.3%), followed by prior training or experience (23.6%) (Table III). Post-operatively, oral NSAIDs were reported to be the most commonly included medication in standard pain management protocol after hip arthroscopy (80.5%) (Table III). Approximately 52% of surgeons included oral non-opioid analgesics, 46% included oral opioids or an intra-articular injection, and 45% included a soft tissue (peri-portal) anesthetic injection. The primary factor influencing choice of pain management protocol after hip arthroscopy was prior training or experience (42.8%), followed by published research (27%) and anesthesiologist preference (20.5%) (Table III). Medications most commonly reported for intra-articular or peri-portal injection after hip arthroscopy were bupivacaine (40.9%), followed by ropivacaine (37.7%) and corticosteroids (13.5%) (Table III). Approximately 21% of respondents reported not using intra-articular or peri-portal injections. The primary factor influencing intra-articular or peri-portal injection choice was prior training (46.5%), followed by published literature (32.1%) and anesthesiologist preference (15.3%) (Table III).

**Table III.** Perioperative pain management

Which of the following medications do you routinely use for pre-emptive analgesia prior to hip arthroscopy? (select all that apply)	RR (%)	What is the primary factor that influences your choice for pre-emptive analgesia?	RR (%)	What is your standard pain management protocol after hip arthroscopy? (select all that apply)	RR (%)	What is the primary factor that influences your choice for your standard pain management protocol after hip arthroscopy?	RR (%)	Which of the following medications do you routinely use for intra-articular or soft tissue (peri-portal) anesthetic injection after hip arthroscopy? (select all that apply)	RR (%)	What is the primary factor that influences your choice for intra-articular or soft tissue (peri-portal) anesthetic injection after hip arthroscopy?	RR (%)
I do not routinely use pre-emptive analgesia prior to hip arthroscopy	41.3	Anesthesiologist preference	45.3	Oral non-steroidal anti-inflammatories (NSAIDs)	80.5	Prior training/experience	42.8	Bupivacaine	40.9	Prior training	46.5
Non-steroidal anti-inflammatories (NSAIDs)	20	Prior Training or experience	23.6	Oral non-opioid analgesic (acetaminophen or similar)	51.6	Published research	27	Ropivacaine	37.7	Published literature	32.1
Non-opioid analgesics (acetaminophen or similar)	19.3	Published research	17.5	Soft tissue (peri-portal) anesthetic injection	45.1	Anesthesiologist preference	20.5	Corticosteroids	13.5	Anesthesiologist preference	15.3
Gabapentinoids	10.8	I do not use pre-emptive analgesia	12.7	Oral opioids	46	Other	9.8	Epinephrine	12.1	Other	6
Opioid medications	8.5	Other	0.5	Intra-articular injection	45.6			Morphine	11.6		
				Fascia iliaca block	7.9			Ketorolac	10.7		
				Femoral nerve block	6.5			Lidocaine	9.8		
				Lumbar plexus block	6			I do not routinely use intra-articular or peri-portal injections	20.9		
				Oral gabapentinoids	3.3						

RR, response rate.

With regards to standard duration of treatment with oral pain medication after hip arthroscopy, the majority of surgeons reported treatment with oral pain medications for 1–2 weeks post-operatively (51.2%) (Table IV). The standard discharge time after surgery was less than 12 h for 42.3% of respondents and between 12 and 24 h for 41.9% of respondents (Table IV). Nearly 75% of respondents reported ‘always’ providing verbal counseling on postoperative pain control after hip arthroscopy (Table IV), as well as providing written instructions on post-operative pain control after surgery (Table IV). Constant or frequent poor pain control after hip arthroscopy was reported by few respondents (7%) (Table IV).

**Table IV.** Postoperative pain management

What is your standard duration of treatment with oral medication for pain after hip arthroscopy?	RR (%)	What is your standard discharge time after hip arthroscopy?	RR (%)	How often do you provide verbal counseling on postoperative pain control after hip arthroscopy?	RR (%)	How often do you provide written instructions on postoperative pain control after hip arthroscopy?	RR (%)	How often do your patients report poor pain control after hip arthroscopy?	RR (%)	In the past year, how often have you referred a patient to a pain management specialist in the first 30 days after a hip arthroscopy?	RR (%)	In the past year, how often have you readmitted a patient to the hospital for pain control in the first 30 days after a hip arthroscopy?	RR (%)	Has any opinion manuscript or expert consensus on clinical/surgical practice affected or directed your care of a patient or patient condition?	RR (%)
Less than 1 week	39.1	Less than 12 h	42.3	Always	74	Always	74.4	Always	3.3	Always	0.5	Always	0.5	Yes	16.8
1–2 weeks	51.2	12–24 h	41.9	Frequently (67–99% of the time)	11.2	Frequently (67–99% of the time)	9.3	Frequently (67–99% of the time)	3.7	Frequently (67–99% of the time)	0.9	Frequently (67–99% of the time)	0.5	No	83.2
Greater than 2 weeks	9.3	Greater than 24 h	15.8	Sometimes (33–67% of the time)	5.6	Sometimes (33–67% of the time)	4.2	Sometimes (33–67% of the time)	12.1	Sometimes (33–67% of the time)	1.9	Sometimes (33–67% of the time)	0.9		
I do not routinely prescribe oral medication for pain after hip arthroscopy	0.9			Infrequently (1–33% of the time)	6	Infrequently (1–33% of the time)	5.1	Infrequently (1–33% of the time)	75.8	Infrequently (1–33% of the time)	16.3	Infrequently (1–33% of the time)	14		
				Never	3.3	Never	7	Never	5.1	Never	80.5	Never	84.2		

RR, response rate.

In the past year, over 80% of respondents denied referring a patient to a pain management specialist in the first 30 days after surgery (Table IV). However, 15.8% of respondents reported readmitting a patient in the past year for pain control in the first 30 days after hip arthroscopy (Table IV). A majority of surgeons (83.2%) reported that opinion manuscripts or expert consensus’ have not influence treatment decisions (Table IV).

### Clinical agreement

Based on the established definitions previously described, the use of oral NSAIDs was the only practice that reached a clinical agreement in the pain management after hip arthroscopy (80.5%). The frequency of providing patients written instructions (74.4%) or verbal counseling (74%) on post-operative pain control after hip arthroscopy achieved general agreement.

### Results and clinical agreement by region

Survey responses to specific pain management questions were divided and analyzed by geographic region to determine differences in regional preferences in pain management after hip arthroscopy. The complete data can be viewed in Supplementary Appendix S1.

In South/Central America, there was general agreement regarding the lack of medication used for pre-emptive analgesia (70.5%) and the inclusion of oral NSAIDs in standard pain management protocols after surgery (69.2%). The standard discharge time of 12–24 h reached a general agreement as well (61.5%). Over 73% of surgeons in South/Central America reported ‘always’ providing verbal counseling on post-operative pain control after surgery.

In North America, there was general agreement in the use of non-opioid analgesics prior to surgery (62.1%). There was a clinical agreement for the inclusion of oral opioids in standard post-operative pain protocol (81.8%), and general agreement for the inclusion of oral NSAIDs (60.6%). Clinical agreement was also reached with regards to discharge time and provision of written instructions for post-operative pain control, as 87.9% of North American participants reported standard discharge time of less than 12 h after surgery and 90.9% reported ‘always’ providing written instructions post-operatively.

In Europe, there was a clinical agreement with the use of oral NSAIDs in standard pain protocol after surgery (80%). General agreement was achieved regarding the use of oral pain medications for 1–2 weeks post-operatively (63.6%).

In Asia/Pacific and the Middle East/Africa, general and clinical achievements were achieved for multiple questions due to the small sample size from each region.

## DISCUSSION

The most important finding of this survey was the absence of clinical agreement and lack of optimization in perioperative pain management following hip arthroscopy. The authors’ hypothesis was confirmed as clinical or general agreement was not met for the majority of survey questions, indicating significant variability in surgeon practice patterns after hip arthroscopy. A clinical agreement of > 80% was only evident in the use of oral NSAIDs after hip arthroscopy. A general agreement of > 60% was only present for the provision of written instructions and verbal counseling on post-operative pain control. It is evident in the survey results that pain management practice patterns after hip arthroscopy can be improved, given the significant number of respondents (15.8%) that had to readmit a patient to the hospital for pain control in the first 30 days after hip arthroscopy in the past year. This fact is especially relevant since the number of hip arthroscopies performed has grown over the past several years and this trend is expected to continue; at the same time, there continues to be an increasing emphasis on outpatient surgery and the use of ambulatory surgery centers [5, 23, 24]. This latter trend is motivated by factors such as greater convenience for families and patients, and a commercial imperative to reduce costs, particularly in North America [2, 25]. Furthermore, readmissions for pain were also observed in fellowship-trained surgeons and those performing a significant number of hip arthroscopies each year. Most of the respondents that reported readmitting a patient for pain control were based in North America (41.1%), were fellowship-trained (67.7%), have been in practice for more than 10 years (64.7%), and reported performing more than 50 hip arthroscopies per year (55.8%). Within this group, most denied routine use of pre-emptive analgesia prior to hip arthroscopy (53%) and relied on prior training and experience (61.8%) as the primary factor influencing the choice of standard pain management protocol after hip arthroscopy. Post-operatively, oral NSAIDs were the most commonly (70.6%) included medication in standard pain management protocol after hip arthroscopy used by this group of respondents, followed by the intra-articular or peri-portal injection of local anesthetics (58.8%), and oral opioids (50%). In addition to its analgesic effect, another possible reason for the popularity of NSAIDs after hip arthroscopy might lie in their beneficial role in reducing the occurrence of heterotopic ossification [26, 27].

A possible explanation for the lack of agreement on most aspects of pain management after hip arthroscopy may be related to factors that influence the surgeons’ choice for the standard pain management protocol. The results of this survey found that only 27% of all respondents use published research as the primary factor to guide their standard pain management protocol, while 63.3% use more personal and anecdotal criteria such as prior training and experience, or anesthesiologist preference, as the primary factor guiding standard pain management protocol. This is consistent with a previous international survey on arthroscopic hip preservation surgery practice patterns demonstrating that surgeons often emulate techniques from their prior training [28]. Further research on the topic of pain management after hip arthroscopy is clearly warranted for the establishment of evidence-based guidelines and the improvement of clinical practice.

Another important finding was the global and geographic differences in the use of oral opioids as a standard of care for pain management after hip arthroscopy. An overwhelming majority of North American respondents (81.8%) reported prescribing oral opioids after hip arthroscopy, compared to respondents in South/Central America (19.2%), Europe (23.6%), Asia/Pacific (44.4%) and Middle East/Africa (20%). The opioid epidemic in the United States is a well-recognized issue [29] and the potential risks of opioid use, in addition to the associated health care costs, should encourage surgeons to seek alternatives to postoperative pain management. The efficacy of non-opioid medications in this setting has been demonstrated in several studies [3, 30, 31] and the American Society of Anesthesiologists (ASA) and the American Pain Society (APS) emphatically recommend the use of non-opioid analgesia in the post-operative period, barring contraindications [32, 33].

Interestingly, North American respondents were also those who most frequently use pre-emptive analgesia. Specifically, 68.2% of North American respondents routinely use pre-emptive analgesia in the management of pain for hip arthroscopy, compared to 29.5% of respondents in South/Central America, 27.3% in Europe, 23.2% in Asia/Pacific and 40% in the Middle East/Africa. Although the discharge time after hip arthroscopy depends on multiple factors, it has been reported that pre-emptive analgesia reduces the time to discharge for a variety of surgical procedures [34, 35].

The survey also found that systemic medications (e.g. oral NSAIDs, oral opioids, oral non-opioids and oral gabapentinoids) were the most commonly reported category of pain management practice after hip arthroscopy. Intra-articular and peri-portal injection of local anesthetics were the second most common category of pain management practiced reported from the survey. The efficacy of this strategy to improve post-operative analgesia and decrease opioid consumption in the perioperative period has been demonstrated in the literature [5, 12]. Shin *et al.* performed a systematic review to identify randomized controlled trials and comparative studies on pain control following hip arthroscopy [5]. Modalities included nerve blocks, peri-portal and intra-articular anesthetic injections and NSAIDs (celecoxib), demonstrating significant variability in postoperative pain management practices within the literature, with limited high-quality evidence. Available data suggested that perioperative analgesia, including nerve blocks, peri-portal and intra-articular injections, and celecoxib may decrease time to discharge and reduce post-operative opioid consumption. However, the authors cautioned that certain modalities, such as peripheral nerve blocks, must be balanced with risks. This may explain why peripheral nerve blocks (fascia iliaca block, femoral nerve block and lumbar plexus block) were the least frequently implemented category of pain management reported by survey respondents, which in previous studies have demonstrated adequate pain relief [5, 14, 16], but may be associated with increased risk of cutaneous nerve deficits and falls [5, 14].

This study should be interpreted in the context of some limitations. First, the inherent sampling bias should be acknowledged, given that the survey was distributed exclusively to members of the medical societies that agreed to collaborate in the study. Furthermore, some surgeons may have been unable to participate given that the questionnaire was only available in the English language; thus, the respondents to the survey may not represent an appropriate sample of the worldwide community of hip arthroscopists. However, given the geographic distribution of respondents, it does not appear that the results were particularly skewed towards English-speaking countries. Second, the questions were designed in a close-ended, multiple-choice format, which prevents the respondents to report responses that did not appear on the questionnaire; however, the questions were designed to contain the most relevant options based on a literature review of the topic. Similarly, the authors acknowledge that other additionally relevant questions were not included, such as those aimed at assessing the proportion of cases performed at outpatient surgery centers, hospitals with same-day discharges, and hospitals with inpatient admissions, given that the setting of surgery plays a significant role in the analgesic decision-making process. Third, hip arthroscopy is used to perform surgeries with different levels of invasiveness, which may account for some variability in pain management practices. Fourth, some of the responses may have been subject to recall bias limiting the accuracy of particular responses (e.g. number of readmissions for pain management, time to discharge, etc.). Lastly, conclusions regarding consensus were also limited by decisions made by the authors for the purposes of data analysis, including the decision to focus on global and regional variability in analgesic practices rather than comparing preferences based on annual case volume or practice type (academic versus private) that may have yielded slightly different results.

## CONCLUSION

There is significant variability in pain management practice after hip arthroscopy. The use of oral NSAIDs in the post-operative period was the only practice that reached a clinical agreement. As the field of hip preservation surgery continues to evolve and expand rapidly, further research on pain management after hip arthroscopy is clearly needed to establish evidence-based guidelines and improve clinical practice.

## SUPPLEMENTARY DATA

Supplementary data are available at *Journal of Hip Preservation Surgery* online.

## Supplementary Material

hnaa050_Supplementary_Data
