# Supplementary material for: Pain management practice patterns after hip arthroscopy: an international survey
Source: J Hip Preserv Surg. 2020 Dec 30;7(3):537–46. doi: 10.1093/jhps/hnaa050 (PMC8081420; doi:10.1093/jhps/hnaa050)
Supplement: hnaa050_Supplementary_Data [file hnaa050_supplementary_data.docx]

**APPENDIX 1**

*South/Central America (n= 78)*

**Table SCA1. How many hip arthroscopies do you perform per year?**

| **Answer Responses** | **Response Rate (%)** |
| --- | --- |
| Less than 20 | 38.5 |
| 20-50 | 35.9 |
| 51-100 | 15.4 |
| 101-200 | 7.7 |
| Greater than 200 | 2.6 |

**Table SCA2. Which of the following medications do you routinely use for preemptive analgesia prior to hip arthroscopy? (select all that apply)**

| **Answer Responses** | **Response Rate (%)** |
| --- | --- |
| I do not routinely use preemptive analgesia prior to hip arthroscopy | 70.5 |
| Non-steroidal anti-inflammatories (NSAIDs) | 17.9 |
| Non-opioid analgesics (acetaminophen or similar) | 12.8 |
| Gabapentinoids | 5.1 |
| Opioid medications | 9 |

**Table SCA3. What is the primary factor that influences your choice for preemptive analgesia?**

| **Answer Responses** | **Response Rate (%)** |
| --- | --- |
| Anesthesiologist preference | 44.9 |
| Prior Training or experience | 19.2 |
| Published research | 17.9 |
| I do not use preemptive analgesia | 17.9 |

**Table SCA4. What is your standard pain management protocol after hip arthroscopy? (select all that apply)**

| **Answer Responses** | **Response Rate (%)** |
| --- | --- |
| Oral non-steroidal anti-inflammatories (NSAIDs) | 69.2 |
| Oral non-opioid analgesic (acetaminophen or similar) | 55.1 |
| Soft tissue (peri-portal) anesthetic injection | 29.5 |
| Oral opioids | 19.2 |
| Intra-articular injection | 39.7 |
| Fascia iliaca block | 3.8 |
| Femoral nerve block | 12.8 |
| Lumbar plexus block | 11.5 |

**Table SCA5. What is the primary factor that influences your choice for your standard pain management protocol after hip arthroscopy?**

| **Answer Responses** | **Response Rate (%)** |
| --- | --- |
| Prior training/experience | 51.3 |
| Published research | 32.1 |
| Anesthesiologist preference | 16.7 |

**Table SCA6. Which of the following medications do you routinely use for intra-articular or soft tissue (peri-portal) anesthetic injection after hip arthroscopy? (select all that apply)**

| **Answer Responses** | **Response Rate (%)** |
| --- | --- |
| Bupivacaine | 38.5 |
| Ropivacaine | 34.6 |
| Corticosteroids | 26.9 |
| Epinephrine | 9 |
| Morphine | 7.7 |
| Ketorolac | 9 |
| Lidocaine | 14.1 |
| I do not routinely use intra-articular or peri-portal injections | 23.1 |

**Table SCA7. What is the primary factor that influences your choice for intra-articular or soft tissue (peri-portal) anesthetic injection after hip arthroscopy?**

| **Answer Responses** | **Response Rate (%)** |
| --- | --- |
| Prior training/experience | 46.2 |
| Published literature | 34.6 |
| Anesthesiologist preference | 14.1 |
| Other | 5.1 |

**Table SCA8. What is your standard duration of treatment with oral medication for pain after hip arthroscopy?**

| **Answer Responses** | **Response Rate (%)** |
| --- | --- |
| Less than 1 week | 41 |
| 1-2 weeks | 47.4 |
| Greater than 2 weeks | 11.5 |

**Table SCA9. What is your standard discharge time after hip arthroscopy?**

| **Answer Responses** | **Response Rate (%)** |
| --- | --- |
| Less than 12 hours | 21.8 |
| 12-24 hours | 61.5 |
| Greater than 24 hours | 16.7 |

**Table SCA10. How often do you provide verbal counseling on postoperative pain control after hip arthroscopy?**

| **Answer Responses** | **Response Rate (%)** |
| --- | --- |
| Always | 73.1 |
| Frequently (67%-99% of the time) | 11.5 |
| Sometimes (33%-67% of the time) | 5.1 |
| Infrequently (1%-33% of the time) | 5.1 |
| Never | 5.1 |

**Table SCA11. How often do you provide written instructions on postoperative pain control after hip arthroscopy?**

| **Answer Responses** | **Response Rate (%)** |
| --- | --- |
| Always | 70.5 |
| Frequently (67%-99% of the time) | 12.8 |
| Sometimes (33%-67% of the time) | 6.4 |
| Infrequently (1%-33% of the time) | 3.8 |
| Never | 6.4 |

**Table SCA12. How often do your patients report poor pain control after hip arthroscopy?**

| **Answer Responses** | **Response Rate (%)** |
| --- | --- |
| Always | 6.4 |
| Frequently (67%-99% of the time) | 6.4 |
| Sometimes (33%-67% of the time) | 16.7 |
| Infrequently (1%-33% of the time) | 64.1 |
| Never | 6.4 |

**Table SCA13. In the past year, how often have you referred a patient to a pain management specialist in the first 30 days after a hip arthroscopy?**

| **Answer Responses** | **Response Rate (%)** |
| --- | --- |
| Always | 1.3 |
| Frequently (67%-99% of the time) | 1.3 |
| Sometimes (33%-67% of the time) | 5.1 |
| Infrequently (1%-33% of the time) | 16.7 |
| Never | 75.6 |

**Table SCA14. In the past year, how often have you readmitted a patient to the hospital for pain control in the first 30 days after a hip arthroscopy?**

| **Answer Responses** | **Response Rate (%)** |
| --- | --- |
| Always | 1.3 |
| Sometimes (33%-67% of the time) | 1.3 |
| Infrequently (1%-33% of the time) | 10.2 |
| Never | 87.2 |

*North America (n= 66)*

**Table NA1. How many hip arthroscopies do you perform per year?**

| **Answer Responses** | **Response Rate (%)** |
| --- | --- |
| Less than 20 | 6.1 |
| 20-50 | 10.6 |
| 51-100 | 28.8 |
| 101-200 | 30.3 |
| Greater than 200 | 24.2 |

**Table NA2. Which of the following medications do you routinely use for preemptive analgesia prior to hip arthroscopy? (select all that apply)**

| **Answer Responses** | **Response Rate (%)** |
| --- | --- |
| I do not routinely use preemptive analgesia prior to hip arthroscopy | 31.8 |
| Non-steroidal anti-inflammatories (NSAIDs) | 47 |
| Non-opioid analgesics (acetaminophen or similar) | 62.1 |
| Gabapentinoids | 39.4 |
| Opioid medications | 25.8 |

**Table NA3. What is the primary factor that influences your choice for preemptive analgesia?**

| **Answer Responses** | **Response Rate (%)** |
| --- | --- |
| Anesthesiologist preference | 36.4 |
| Prior Training or experience | 25.8 |
| Published research | 24.2 |
| I do not use preemptive analgesia | 6.1 |
| Other | 7.6 |

**Table NA4. What is your standard pain management protocol after hip arthroscopy? (select all that apply)**

| **Answer Responses** | **Response Rate (%)** |
| --- | --- |
| Oral non-steroidal anti-inflammatories (NSAIDs) | 71.2 |
| Oral non-opioid analgesic (acetaminophen or similar) | 48.5 |
| Soft tissue (peri-portal) anesthetic injection | 60.6 |
| Oral opioids | 81.8 |
| Intra-articular injection | 45.5 |
| Fascia iliaca block | 16.7 |
| Lumbar plexus block | 4.5 |

**Table NA5. What is the primary factor that influences your choice for your standard pain management protocol after hip arthroscopy?**

| **Answer Responses** | **Response Rate (%)** |
| --- | --- |
| Prior training/experience | 53 |
| Published research | 37.9 |
| Anesthesiologist preference | 6.1 |
| Other | 3 |

**Table NA6. Which of the following medications do you routinely use for intra-articular or soft tissue (peri-portal) anesthetic injection after hip arthroscopy? (select all that apply)**

| **Answer Responses** | **Response Rate (%)** |
| --- | --- |
| Bupivacaine | 42.4 |
| Ropivacaine | 50 |
| Epinephrine | 19.7 |
| Morphine | 21.2 |
| Ketorolac | 12.1 |
| Lidocaine | 7.6 |
| I do not routinely use intra-articular or peri-portal injections | 12.1 |

**Table NA7. What is the primary factor that influences your choice for intra-articular or soft tissue (peri-portal) anesthetic injection after hip arthroscopy?**

| **Answer Responses** | **Response Rate (%)** |
| --- | --- |
| Prior training | 51.5 |
| Published literature | 37.9 |
| Anesthesiologist preference | 6.1 |
| Other | 4.5 |

**Table NA8. What is your standard duration of treatment with oral medication for pain after hip arthroscopy?**

| **Answer Responses** | **Response Rate (%)** |
| --- | --- |
| Less than 1 week | 54.5 |
| 1-2 weeks | 40.9 |
| Greater than 2 weeks | 4.5 |

**Table NA9. What is your standard discharge time after hip arthroscopy?**

| **Answer Responses** | **Response Rate (%)** |
| --- | --- |
| Less than 12 hours | 87.9 |
| 12-24 hours | 9.1 |
| Greater than 24 hours | 3 |

**Table NA10. How often do you provide verbal counseling on postoperative pain control after hip arthroscopy?**

| **Answer Responses** | **Response Rate (%)** |
| --- | --- |
| Always | 86.3 |
| Frequently (67%-99% of the time) | 9.1 |
| Sometimes (33%-67% of the time) | 3 |
| Infrequently (1%-33% of the time) | 1.5 |

**Table NA11. How often do you provide written instructions on postoperative pain control after hip arthroscopy?**

| **Answer Responses** | **Response Rate (%)** |
| --- | --- |
| Always | 90.9 |
| Frequently (67%-99% of the time) | 6.1 |
| Infrequently (1%-33% of the time) | 1.5 |
| Never | 1.5 |

**Table NA12. How often do your patients report poor pain control after hip arthroscopy?**

| **Answer Responses** | **Response Rate (%)** |
| --- | --- |
| Always | 1.5 |
| Sometimes (33%-67% of the time) | 3 |
| Infrequently (1%-33% of the time) | 89.4 |
| Never | 6.1 |

**Table NA13. In the past year, how often have you referred a patient to a pain management specialist in the first 30 days after a hip arthroscopy?**

| **Answer Responses** | **Response Rate (%)** |
| --- | --- |
| Frequently (67%-99% of the time) | 1.5 |
| Infrequently (1%-33% of the time) | 16.7 |
| Never | 81.8 |

**Table NA14. In the past year, how often have you readmitted a patient to the hospital for pain control in the first 30 days after a hip arthroscopy?**

| **Answer Responses** | **Response Rate (%)** |
| --- | --- |
| Sometimes (33%-67% of the time) | 1.5 |
| Infrequently (1%-33% of the time) | 19.7 |
| Never | 78.8 |

*Europe (n= 55)*

**Table E1. How many hip arthroscopies do you perform per year?**

| **Answer Responses** | **Response Rate (%)** |
| --- | --- |
| Less than 20 | 16.4 |
| 20-50 | 18.2 |
| 51-100 | 32.7 |
| 101-200 | 20 |
| Greater than 200 | 12.7 |

**Table E2. Which of the following medications do you routinely use for preemptive analgesia prior to hip arthroscopy? (select all that apply)**

| **Answer Responses** | **Response Rate (%)** |
| --- | --- |
| I do not routinely use preemptive analgesia prior to hip arthroscopy | 72.7 |
| Non-steroidal anti-inflammatories (NSAIDs) | 23.6 |
| Non-opioid analgesics (acetaminophen or similar) | 12.7 |
| Gabapentinoids | 5.5 |

**Table E3. What is the primary factor that influences your choice for preemptive analgesia?**

| **Answer Responses** | **Response Rate (%)** |
| --- | --- |
| Anesthesiologist preference | 54.5 |
| Prior Training/experience | 27.3 |
| Published research | 5.5 |
| I do not use preemptive analgesia | 12.7 |

**Table E4. What is your standard pain management protocol after hip arthroscopy? (select all that apply)**

| **Answer Responses** | **Response Rate (%)** |
| --- | --- |
| Oral non-steroidal anti-inflammatories (NSAIDs) | 80 |
| Oral non-opioid analgesic (acetaminophen or similar) | 47.3 |
| Soft tissue (peri-portal) anesthetic injection | 43.6 |
| Oral opioids | 23.6 |
| Intra-articular injection | 49.1 |
| Femoral nerve block | 5.5 |
| Lumbar plexus block | 1.8 |

**Table E5. What is the primary factor that influences your choice for your standard pain management protocol after hip arthroscopy?**

| **Answer Responses** | **Response Rate (%)** |
| --- | --- |
| Prior training/experience | 45.5 |
| Published research | 9.1 |
| Anesthesiologist preference | 43.6 |

**Table E6. Which of the following medications do you routinely use for intra-articular or soft tissue (peri-portal) anesthetic injection after hip arthroscopy? (select all that apply)**

| **Answer Responses** | **Response Rate (%)** |
| --- | --- |
| Bupivacaine | 36.4 |
| Corticosteroids | 7.3 |
| Epinephrine | 5.5 |
| Morphine | 3.6 |
| Ketorolac | 5.5 |
| Lidocaine | 7.3 |
| I do not routinely use intra-articular or peri-portal injections | 30.9 |

**Table E7. What is the primary factor that influences your choice for intra-articular or soft tissue (peri-portal) anesthetic injection after hip arthroscopy?**

| **Answer Responses** | **Response Rate (%)** |
| --- | --- |
| Prior training/experience | 49.1 |
| Published literature | 20 |
| Anesthesiologist preference | 29.1 |
| Other | 1.8 |

**Table E8. What is your standard duration of treatment with oral medication for pain after hip arthroscopy?**

| **Answer Responses** | **Response Rate (%)** |
| --- | --- |
| Less than 1 week | 21.8 |
| 1-2 weeks | 63.6 |
| Greater than 2 weeks | 12.7 |
| I do not routinely prescribe oral medication for pain after hip arthroscopy | 1.8 |

**Table E9. What is your standard discharge time after hip arthroscopy?**

| **Answer Responses** | **Response Rate (%)** |
| --- | --- |
| Less than 12 hours | 23.6 |
| 12-24 hours | 45.5 |
| Greater than 24 hours | 30.9 |

**Table E10. How often do you provide verbal counseling on postoperative pain control after hip arthroscopy?**

| **Answer Responses** | **Response Rate (%)** |
| --- | --- |
| Always | 58.2 |
| Frequently (67%-99% of the time) | 14.5 |
| Sometimes (33%-67% of the time) | 9.1 |
| Infrequently (1%-33% of the time) | 14.5 |
| Never | 3.6 |

**Table E11. How often do you provide written instructions on postoperative pain control after hip arthroscopy?**

| **Answer Responses** | **Response Rate (%)** |
| --- | --- |
| Always | 65.4 |
| Frequently (67%-99% of the time) | 9.1 |
| Sometimes (33%-67% of the time) | 7.3 |
| Infrequently (1%-33% of the time) | 9.1 |
| Never | 9.1 |

**Table E12. How often do your patients report poor pain control after hip arthroscopy?**

| **Answer Responses** | **Response Rate (%)** |
| --- | --- |
| Always | 1.8 |
| Frequently (67%-99% of the time) | 5.5 |
| Sometimes (33%-67% of the time) | 16.4 |
| Infrequently (1%-33% of the time) | 74.5 |
| Never | 1.8 |

**Table E13. In the past year, how often have you referred a patient to a pain management specialist in the first 30 days after a hip arthroscopy?**

| **Answer Responses** | **Response Rate (%)** |
| --- | --- |
| Infrequently (1%-33% of the time) | 18.2 |
| Never | 81.8 |

**Table E14. In the past year, how often have you readmitted a patient to the hospital for pain control in the first 30 days after a hip arthroscopy?**

| **Answer Responses** | **Response Rate (%)** |
| --- | --- |
| Frequently (67%-99% of the time) | 1.8 |
| Infrequently (1%-33% of the time) | 14.5 |
| Never | 83.6 |

*Asia/Pacific (n= 9)*

**Table AP1. How many hip arthroscopies do you perform per year?**

| **Answer Responses** | **Response Rate (%)** |
| --- | --- |
| Less than 20 | 33.3 |
| 20-50 | 33.3 |
| 51-100 | 22.2 |
| Greater than 200 | 11.1 |

**Table AP2. Which of the following medications do you routinely use for preemptive analgesia prior to hip arthroscopy? (select all that apply)**

| **Answer Responses** | **Response Rate (%)** |
| --- | --- |
| I do not routinely use preemptive analgesia prior to hip arthroscopy | 77.8 |
| Non-steroidal anti-inflammatories (NSAIDs) | 11.1 |
| Opioid medications | 11.1 |

**Table AP3. What is the primary factor that influences your choice for preemptive analgesia?**

| **Answer Responses** | **Response Rate (%)** |
| --- | --- |
| Anesthesiologist preference | 44.4 |
| Prior Training or experience | 11.1 |
| Published research | 33.3 |
| I do not use preemptive analgesia | 11.1 |

**Table AP4. What is your standard pain management protocol after hip arthroscopy? (select all that apply)**

| **Answer Responses** | **Response Rate (%)** |
| --- | --- |
| Oral non-steroidal anti-inflammatories (NSAIDs) | 77.8 |
| Oral non-opioid analgesic (acetaminophen or similar) | 66.7 |
| Soft tissue (peri-portal) anesthetic injection | 44.4 |
| Oral opioids | 44.4 |
| Intra-articular injection | 77.8 |

**Table AP5. What is the primary factor that influences your choice for your standard pain management protocol after hip arthroscopy?**

| **Answer Responses** | **Response Rate (%)** |
| --- | --- |
| Prior training/experience | 44.4 |
| Published research | 33.3 |
| Anesthesiologist preference | 11.1 |
| Other | 11.1 |

**Table AP6. Which of the following medications do you routinely use for intra-articular or soft tissue (peri-portal) anesthetic injection after hip arthroscopy? (select all that apply)**

| **Answer Responses** | **Response Rate (%)** |
| --- | --- |
| Bupivacaine | 44.4 |
| Corticosteroids | 22.2 |
| Epinephrine | 11.1 |
| Morphine | 22.2 |
| Ketorolac | 22.2 |
| I do not routinely use intra-articular or peri-portal injections | 22.2 |

**Table AP7. What is the primary factor that influences your choice for intra-articular or soft tissue (peri-portal) anesthetic injection after hip arthroscopy?**

| **Answer Responses** | **Response Rate (%)** |
| --- | --- |
| Prior training | 77.8 |
| Published literature | 22.2 |

**Table AP8. What is your standard duration of treatment with oral medication for pain after hip arthroscopy?**

| **Answer Responses** | **Response Rate (%)** |
| --- | --- |
| Less than 1 week | 22.2 |
| 1-2 weeks | 66.7 |
| Greater than 2 weeks | 11.1 |

**Table AP9. What is your standard discharge time after hip arthroscopy?**

| **Answer Responses** | **Response Rate (%)** |
| --- | --- |
| 12-24 hours | 77.8 |
| Greater than 24 hours | 22.2 |

**Table AP10. How often do you provide verbal counseling on postoperative pain control after hip arthroscopy?**

| **Answer Responses** | **Response Rate (%)** |
| --- | --- |
| Always | 77.8 |
| Sometimes (33%-67% of the time) | 11.1 |
| Never | 11.1 |

**Table AP11. How often do you provide written instructions on postoperative pain control after hip arthroscopy?**

| **Answer Responses** | **Response Rate (%)** |
| --- | --- |
| Always | 55.6 |
| Infrequently (1%-33% of the time) | 11.1 |
| Never | 33.3 |

**Table AP12. How often do your patients report poor pain control after hip arthroscopy?**

| **Answer Responses** | **Response Rate (%)** |
| --- | --- |
| Sometimes (33%-67% of the time) | 22.2 |
| Infrequently (1%-33% of the time) | 66.7 |
| Never | 11.1 |

**Table AP13. In the past year, how often have you referred a patient to a pain management specialist in the first 30 days after a hip arthroscopy?**

| **Answer Responses** | **Response Rate (%)** |
| --- | --- |
| Never | 100 |

**Table AP14. In the past year, how often have you readmitted a patient to the hospital for pain control in the first 30 days after a hip arthroscopy?**

| **Answer Responses** | **Response Rate (%)** |
| --- | --- |
| Never | 100 |

*Middle East/Africa (n= 5)*

**Table MEA1. How many hip arthroscopies do you perform per year?**

| **Answer Responses** | **Response Rate (%)** |
| --- | --- |
| Less than 20 | 20 |
| 51-100 | 20 |
| 101-200 | 60 |

**Table MEA2. Which of the following medications do you routinely use for preemptive analgesia prior to hip arthroscopy? (select all that apply)**

| **Answer Responses** | **Response Rate (%)** |
| --- | --- |
| I do not routinely use preemptive analgesia prior to hip arthroscopy | 60 |
| Non-steroidal anti-inflammatories (NSAIDs) | 20 |
| Non-opioid analgesics (acetaminophen or similar) | 20 |
| Opioid medications | 20 |

**Table MEA3. What is the primary factor that influences your choice for preemptive analgesia?**

| **Answer Responses** | **Response Rate (%)** |
| --- | --- |
| Anesthesiologist preference | 60 |
| Published research | 40 |

**Table MEA4. What is your standard pain management protocol after hip arthroscopy? (select all that apply)**

| **Answer Responses** | **Response Rate (%)** |
| --- | --- |
| Oral non-steroidal anti-inflammatories (NSAIDs) | 60 |
| Oral non-opioid analgesic (acetaminophen or similar) | 60 |
| Oral opioids | 20 |
| Intra-articular injection | 60 |
| Fascia iliaca block | 60 |
| Femoral nerve block | 20 |

**Table MEA5. What is the primary factor that influences your choice for your standard pain management protocol after hip arthroscopy?**

| **Answer Responses** | **Response Rate (%)** |
| --- | --- |
| Prior training/experience | 40 |
| Published research | 20 |
| Anesthesiologist preference | 40 |

**Table MEA6. Which of the following medications do you routinely use for intra-articular or soft tissue (peri-portal) anesthetic injection after hip arthroscopy? (select all that apply)**

| **Answer Responses** | **Response Rate (%)** |
| --- | --- |
| Bupivacaine | 80 |
| Ropivacaine | 20 |
| Corticosteroids | 20 |
| Epinephrine | 20 |
| Ketorolac | 40 |

**Table MEA7. What is the primary factor that influences your choice for intra-articular or soft tissue (peri-portal) anesthetic injection after hip arthroscopy?**

| **Answer Responses** | **Response Rate (%)** |
| --- | --- |
| Published literature | 60 |
| Anesthesiologist preference | 40 |

**Table MEA8. What is your standard duration of treatment with oral medication for pain after hip arthroscopy?**

| **Answer Responses** | **Response Rate (%)** |
| --- | --- |
| 1-2 weeks | 100 |

**Table MEA9. What is your standard discharge time after hip arthroscopy?**

| **Answer Responses** | **Response Rate (%)** |
| --- | --- |
| Less than 12 hours | 20 |
| 12-24 hours | 80 |

**Table MEA10. How often do you provide verbal counseling on postoperative pain control after hip arthroscopy?**

| **Answer Responses** | **Response Rate (%)** |
| --- | --- |
| Always | 80 |
| Frequently (67%-99% of the time) | 20 |

**Table MEA11. How often do you provide written instructions on postoperative pain control after hip arthroscopy?**

| **Answer Responses** | **Response Rate (%)** |
| --- | --- |
| Always | 40 |
| Frequently (67%-99% of the time) | 20 |
| Infrequently (1%-33% of the time) | 20 |
| Never | 20 |

**Table MEA12. How often do your patients report poor pain control after hip arthroscopy?**

| **Answer Responses** | **Response Rate (%)** |
| --- | --- |
| Infrequently (1%-33% of the time) | 100 |

**Table MEA13. In the past year, how often have you referred a patient to a pain management specialist in the first 30 days after a hip arthroscopy?**

| **Answer Responses** | **Response Rate (%)** |
| --- | --- |
| Infrequently (1%-33% of the time) | 20 |
| Never | 80 |

**Table MEA14. In the past year, how often have you readmitted a patient to the hospital for pain control in the first 30 days after a hip arthroscopy?**

| **Answer Responses** | **Response Rate (%)** |
| --- | --- |
| Infrequently (1%-33% of the time) | 20 |
| Never | 80 |
